# Effects of Royal Jelly and Tocotrienol Rich Fraction in obesity treatment of calorie-restricted obese rats: a focus on white fat browning properties and thermogenic capacity

**DOI:** 10.1186/s12986-020-00458-8

**Published:** 2020-06-01

**Authors:** Naimeh Mesri Alamdari, Pardis Irandoost, Neda Roshanravan, Mohammadreza Vafa, Mohammad Asghari Jafarabadi, Shahriar Alipour, Leila Roshangar, Mohammadreza Alivand, Farnaz Farsi, Farzad Shidfar

**Affiliations:** 1 Department of Nutrition,Student research committee, School of Health, Iran University of Medical Sciences, Tehran, Iran; 2 Cardiovascular Research Center, Tabriz University of Medical Sciences, Tabriz, Iran; 3 Road Traffic Injury Prevention Research Center, School of Health, Tabriz University of Medical Sciences, Tabriz, Iran; 4 Department of Biochemistry, Faculty of Medicine, Urmia University of Medical Sciences, Urmia, Iran; 5 Stem Cell Research Center, Tabriz University of Medical Sciences, Tabriz, Iran; 6 Department of Medical Genetics, Faculty of Medicine, Tabriz University of Medical Sciences, Tabriz, Iran; 7 Colorectal Research Center, Iran University of medical sciences, Tehran, Iran; 8 Department of Nutrition, School of Health, Iran University of Medical Sciences, Tehran, Iran

**Keywords:** Obesity, Calorie restriction, White adipose tissue, Brown adipose tissue, Royal jelly, Tocotrienol rich fraction

## Abstract

**Background:**

Obesity has reached an alarming rate worldwide. Promoting thermogenesis via increasing the function of brown adipose tissue (BAT) or white adipose tissue (WAT) browning has been proposed as a new protective approach against obesity. The goal of this study was to evaluate the effects of Royal Jelly (RJ) and tocotrienol rich fraction (TRF) on BAT activation and WAT browning during calorie restriction diet (CRD) in obesity model.

**Methods:**

In this experimental study, 50 obese Wistar rats were randomly divided into 5 groups and then received one of the following treatments for a period of 8-week: High-fat diet (HFD), CRD, RJ + CRD, TRF + CRD, and RJ + TRF + CRD. Effects of RJ and TRF, individually and in combination on body weight and the expression of key thermoregulatory genes in WAT and BAT were examined by quantitative real-time (qRT-PCR). Also, morphological alterations were assessed by hematoxylin and eosin staining.

**Results:**

RJ (− 67.21 g ±4.84 g) and RJ + TRF (− 73.29 g ±4.51 g) significantly reduced weight gain relative to the CRD group (− 40.70 g ±6.50 g, *P* < 0.001). In comparison with the CRD group, RJ and RJ + TRF remarkably enhanced the uncoupling protein1 *(UCP1)* expression in WAT (5.81, 4.72 fold, *P* < 0.001) and BAT (4.99, 4.75 fold, *P* < 0.001). The expression of PR domain containing 16*(PRDM 16)*, cAMP response element-binding protein1 *(CREB1)*, P38 mitogen-activated protein kinases *(P38MAPK),* and Bone morphogenetic protein8B *(BMP8B)* have significantly increased following RJ and RJ + TRF treatments (*P* < 0.001). However**,** the expression levels of CCAAT/enhancer-binding protein beta *(CEBPβ)* and Bone morphogenetic protein7 *(BMP7)* did not remarkably change. Multilocular beige cells in WAT and compacted dense adipocytes were also observed in BAT of RJ and RJ + TRF received groups. TRF showed no substantial effects on the expression of the mentioned thermoregulatory genes and brown fat-like phenotype.

**Conclusion:**

Our results suggest that, Royal Jelly promotes thermogenesis and browning of WAT, contributing to an increase in energy expenditure. Thus, Royal Jelly may give rise to a novel dietary choice to attenuate obesity.

## Introduction

The expanding obesity rate worldwide arises from the complex interactions among the environmental factors, genetic context, and individual behaviors. Nonetheless, the disproportion in energy intake, and energy expenditure is thought to be the most determining aspect of obesity [1]. Although calorie restriction is the primary intervention in obesity management, it seems to be an inefficient approach in long-term, since metabolic adaptations accrue in response to energy limitation, which may result from reductions in thermogenesis, resting energy expenditure or other energy expenditure constituents [2–4].

Unlike white adipose tissue (WAT), which is the main site of excess energy; brown adipose tissue is a primary site for adaptive thermogenesis. The thermogenic capacity of brown adipocytes relies mostly on the high expression of Uncoupling protein1 (UCP1) *and* high mitochondrial content. When activated, mediates chemical energy dissipation through dissociation of mitochondrial substrate oxidation from Adenosine triphosphate (ATP) production, which resulted in the generation of heat [5, 6]. Hence, Brown adipose tissue (BAT) function and activation have substantial potential attention from a therapeutic perspective, its vital function in obesity control is also remarkable.

In addition to classical BAT, a phenomenon called “browning or beigeing procedure” has been demonstrated including the development of brite adipocytes in classical WAT [7–9]. It is suggested that, some stimulants such as cold exposure, β-adrenergic receptor stimuli, exercise, PPARs agonists, pharmacological agents, and some food components may develop brite or beige adipocytes [1, 10]. Browning of WAT protects against obesity through increasing energy consumption, which can lead to a negative energy balance [1, 7]. It is speculated that, PR domain containing16 *(PRDM16),* Bone morphogenetic proteins *(BMPs)* and CCAAT/enhancer-binding protein beta (*C/EBPβ)* are the master regulators, which their interactions participate in the *UCP1* gene regulation that ultimately contribute to BAT activation or WAT remodeling [11–14]. Identification of the food components that can induce the browning represents as an attractive potential way in obesity treatment. Royal Jelly (RJ) is a yellowish-white, multifunctional creamy material secreted from the hypopharyngeal and mandibular glands of nurse honeybees [15]. The main components of RJ are 10-hydroxy-Trans- 2-decenoic acid (HDEA) and Hydroxydecanoic acid ( HDAA), and main biological activities of RJ attribute to them [16]. RJ has multiple biological functions such as antioxidant, antitumor, antiaging, antihypercholesterolemic, anti-inflammatory, antimicrobial, hypoglycemic, radio-protective, gastro-protective, hepato-protective, and vasodilative effects [17]. RJ caused a remarkable decrease in body weight and abdominal fat depots and also an increase in skeletal muscle mass in High-fat diet (HFD) induced obese rats [18]. Nevertheless, the exact effects of RJ on the regulation of thermogenesis and browning of white adipose tissue have not been defined yet, as well as the procedure by which RJ ameliorate obesity is not exactly figured out. Vitamin E is a lipid-soluble nutrient, composed of two biologically active Tocopherols (TP) and Tocotrienols (T3) subclasses with eight analogs each one including α-, β-, γ-, and δ-forms [19]. Most of the studies on vitamin E have concentrated on TPs; therefore, very little is known about T3s. Main food resources of T3s are rice bran, oat, wheat germ, palm oil, and annatto oil. T3s have been indicated to possess various physiological activities including neuroprotective, anticancer, antiangiogenesis, anti-tumor, cardiovascular-protective, hypocholesterolemic, and anti-inflammatory properties [20, 21]. However, T3s effect on obesity management and its related metabolic challenges have been less assessed up to now, compared with saturated matched TPs, and also the chief mechanisms of action in obesity regulation are still unknown [19]. Gamma-tocotrienol is the most common T3 isomer and is physiologically more available compared to other isomers [22]. By considering the unsuccessful methods of obesity control and the adverse consequence of long-term calorie restriction on energy expenditure and thermogenesis, applying functional foods with the properties of thermogenesis improvement would be profitable. Up to the best of our knowledge, the potential effects of royal jelly and γ-tocotrienol on white fat browning and thermogenesis induction during calorie restriction diet has not been examined yet. Thus, the objective of this study was to evaluate the effect of royal jelly, γ-tocotrienol, and their combinations on the induction of genes involved in the beige phenotype appearance in WAT and also BAT activation using molecular involved mechanisms in obesity models of rats during calorie restriction diet ( CRD).

## Method

### Animals and treatments

In this experimental investigation, 55 Male Wistar rats (3 weeks old) weighing 50–70 g were purchased from Pasteur Institute (Tehran, Iran). All rats were kept individually in stainless steel cages under the standard condition temperature of 22–25 °C and relative humidity 55 ± 5%, with a 12-h light/dark cycle (7:00–19:00 h), allowed free access to water and a normal chow diet for 1 week. All experimental procedures performed on animals complied with the National Institutes of Health guide for the care and use of laboratory animals [23] and approved by the Ethics Committee of Iran University of medical science (ethic code: IR.IUMS.FMD.REC 1396.9321324003). All efforts were made to decrease the sample size of studied rats and minimized animal suffering.

**Fig. 1:**
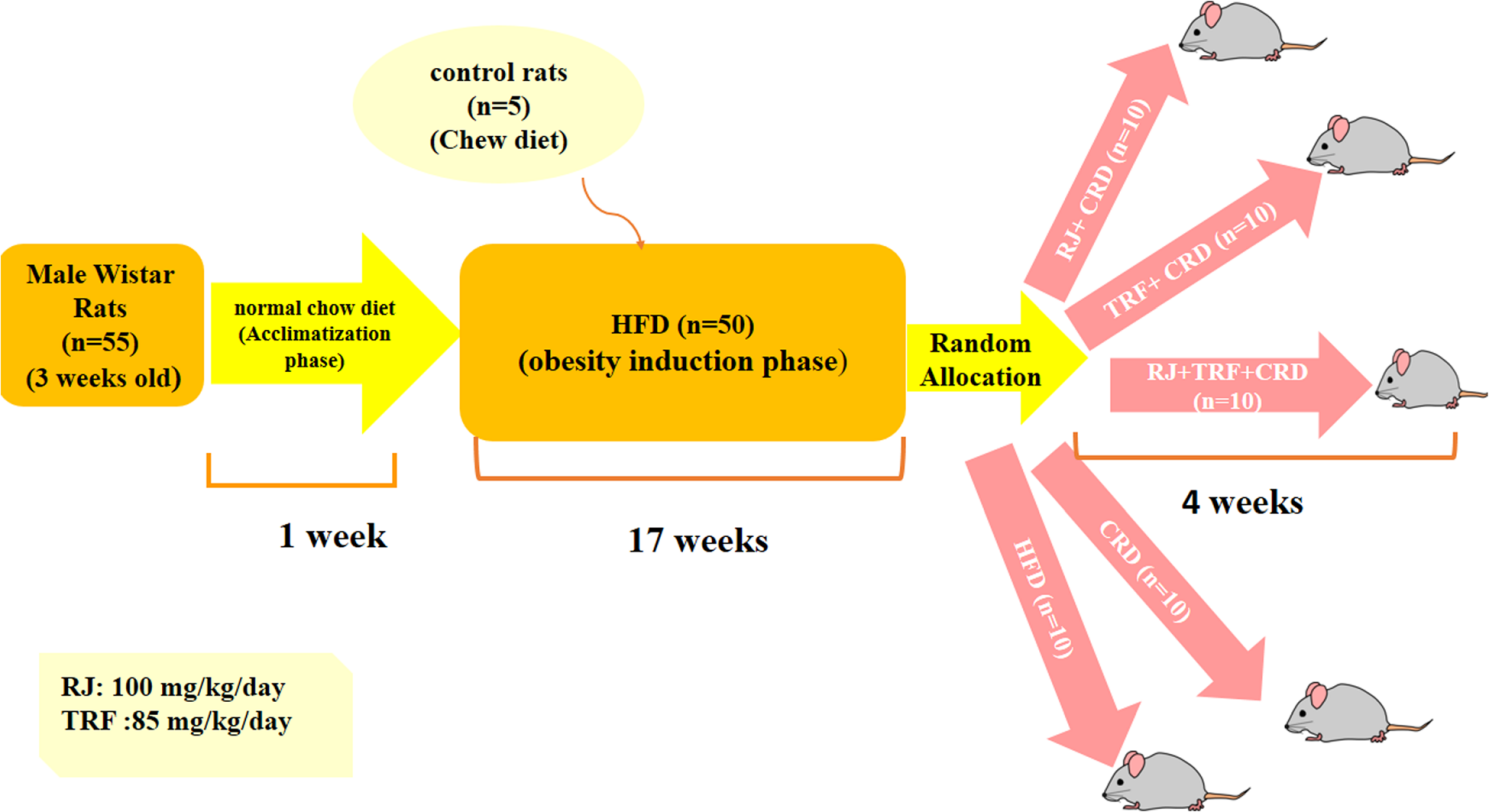
Scheme of study design

The study protocol consisted of a two-phase 1) obesity induction period 2) treatment period. (Fig. 1) After 1 week of acclimatization, 50 rats were administered a HFD to induce the obesity model and five rats received normal chow diet as the control group for HFD receiving rats. All rats had free access to food and water in this phase of the study. Semi-purified HFD consisted of standard chow powder mixed with milk butter (40% w/w). The compositions of the diets used in the study are shown in Table 1. HFD was prepared every 2 days freshly in the form of pellets and kept at 4 °C to maintain nutrients. We weighted animals every week. At the end of the 17th weeks, the mean weight of HFD administered rats increased significantly compared to normal chow diet consuming rats (443.28 g ± 46.62 g vs 396.24 g ±28.79 g *P* < 0.05), indicating that HFD induced obesity model was accomplished. At the second phase, rats were randomly allocated to one of 5 groups (*n* = 10/group) using a randomized block procedure which matched for body weight and treated for 8 weeks as follows 1) RJ group receiving lyophilized RJ powder (100 mg/kg/day) orally dissolved in CRD 2) Tocotrienol rich fraction (TRF) group receiving TRF (85 mg/kg/day) orally dissolved in CRD 3) RJ + TRF group receiving both 100 mg/kg/day lyophilized RJ powder and 85 mg/kg/day TRF orally dissolved in CRD 4) CRD group; without any supplementation as control for RJ, TRF and RJ + TRF groups and **5)** HFD group; HFD without any supplementation as control for CRD group.

**Table 1:** Composition of the experimental diets

Dietary composition (g/kg)	Chow	HFD
Carbohydrate	**536.2**	**335.125**
Fiber	**42**	**26.25**
Protein	**260.8**	**163**
Lipid	**40**	**400**
Calcium	**9.5**	**5.93**
Phosphorus	**6.5**	**4.06**
Salt	**5**	**3.125**
Moisture	**50**	**31.25**
Ash	**50**	**31.25**
Energy density (kcal/g)	**3.6**	**5.6**

*HFD* high-fat diet

CRD had the same macronutrient composition as HFD (Table 1), but the calorie content was 30% lower than the ad libitum intake of HFD. RJ and TRF were added to CRD and the food was weighed, then given to rats every day in certain time schedules (between 9:00–9:30 AM).HFD was fed ad libitum and given to rats every day.

Lyophilized Royal jelly powder was purchased from Bulk Supplements Co, Ltd., (Henderson, USA) containing 6% of 10-HAD. TRF was kindly provided by ExcelVite Co, Ltd. (Perak, Malaysia).High-performance liquid chromatography determined that TRF contained α-tocotrienol (12%), β-tocotrienol (2%), γ-tocotrienol (19.3%) and δ-tocotrienol (5.5%) together with α-tocopherol (11.9%). The doses and duration of treatments were selected based on the previously reported oral no-observed-adverse-effects and the sample size was decided based on similar work done before [19, 24].

### Sample collection

At the end of the study, all rats were anesthetized with intraperitoneal injection of xylazine (xylazine 2%, 20 mg ml − 1, Alfasan, Woerden, Netherlands) and ketamine (ketamine 10%, 100 mg ml − 1, Alfasan, Woerden, Netherlands) after overnight fasting and interscapular BAT, inguinal WAT and hypothalamus were quickly removed, rinsed gently with phosphate-buffered saline (PBS) solution, and kept in RNA later Stabilization Solution (Qiagen, Inc. Germantown, Maryland, USA) f or RNA isolation.

### Quantitative real-time PCR

All tissues were homogenized gently. Total RNA was extracted from each tissue using Trizol (Thermo Fisher, Waltham, Massachusetts, USA) according to the manufacturer’s protocol. The quality and quantity of extracted RNA were determined spectrophotometrically, measuring relative absorbance ratio at A260/280 and A260/230 (NanoDrop One/Once, Thermo Fisher Scientific Inc., Wilmington, Delaware, USA).

The extracted RNA was converted to Complementary DNA (cDNA) using the RevertAid First Strand cDNA Synthesis Kit (Thermo Scientific, Waltham, Massachusetts, USA) with 1 μg of mRNA according to the manufacturer’s protocol. The real-time reverse transcription-polymerase chain reaction (RT-PCR) was done using a fluorescence thermal cycler (Light Cycler system; Roche Diagnostics, Mannheim, Germany) system using SYBR Premix Ex Taq (Takara Bio Inc., Shiga, Japan) and gene-specific primers for cAMP response element-binding protein1*(CREB1)*P38 mitogen-activated protein kinases *(P38MAPK)* Bone morphogenetic protein7 *(BMP7)*, Bone morphogenetic protein8B*(BMP8B), C/EBP β, PRDM16, UCP1*, and *β-actin*. The primer sequences were designed through the reported sequences of Primer Bank NCBI, summarized in Table 2 and obtained from Metabion international AG (Steinkirchen, Germany). Delta-delta method was used to calculate the relative mRNA expression of the target gene and normalized to β-Actin as a reference gene [25]. PCR was done under the following conditions: 95 °C for 10 min, 95 °C for 10 s, and 60 °C for 10 s for 45 cycles with 100% ramp rate under standard conditions. Triplicate Ct values were calculated for each sample.

**Table 2:** Sequences of primers used for qRT-PCR in study

Gene	Forward	Reverse
**CREB-1**	**CTACAATATGCACAGACCACT**	**GAGGACGCCATAACAACTCCA**
**p38MAPK**	**GACCTAAAGCCCAGCAACCTC**	**CGTAGCCGGTCATTTCGTCA**
**C/EBP β**	**ACACGGGACTGACGCAAC**	**AAACATCAACAGCAACAACCC**
**PRDM16**	**CCAAAACCGTGTGATAAGGTC**	**GGGTATTTGGCACATTAACAAC**
**BMP7**	**TTCCTCACCGACGCCGACA**	**AAGATCAAACCGGAACTCTCGAT**
**BMP8B**	**TCGAGCACCACTAGCGACT**	**GTTGCCACTGTCATCCGTCA**
**UCP-1**	**TTCTTTTCTGCGACTCGGAT**	**GCCCAATGGTGTTTAGCATC**
**β-actin**	**TCAGGTCATCACTATCGGCAA**	**TTACGGATGTCAACGTCACAC**

### Histological assay

The interscapular BAT and inguinal WAT of randomly selected two rats from each studied group were removed, rinsed gently with PBS solution, then fixed in 10% buffered formalin with the change of formalin every 2 days for 7 days. The samples were then dehydrated through different solutions of alcohol and then paraffin-embedded. Tissues were cut by rotary microtome in thin sections. For histological studies, 5 μm thick sections stained with the H&E method and studied with a Nikon light microscope. For histomorphometric studies, adipose tissue assessment was carried out according to previous studies [26] briefly from each adipose specimen, in 10 randomly selected microscopic fields, a total of 100 crosses sectioned adipose tissue were analyzed and the percentage of each parameter which contained; three types of adipose tissue and connective tissue were averaged for each group using 40X objective lens. One experienced histologist who was blinded to treatment groups assessed the histological examination. Each experiment was performed in triplicate.

### Statistical analysis

The normality of data was assessed by the one-sample Kolmogorov-Smirnov test. All data were represented as the mean ± SEM. One-way analysis of variance (ANOVA) was done to test the differences between groups. Tukey’s post hoc was performed to analyze the multiple comparisons.

IBM SPSS Statistics 23 (IBM SPSS Statistics, Armonk, USA) was applied to analyze all data.

Figures were visualized using the Prism software, version 8·0 (GraphPad, CA, USA). A significant level was considered as *P*-value < 0.05.

## Results

### Effects of CRD, RJ, TRF and mixed treatments on weight changes

**Fig. 2:**
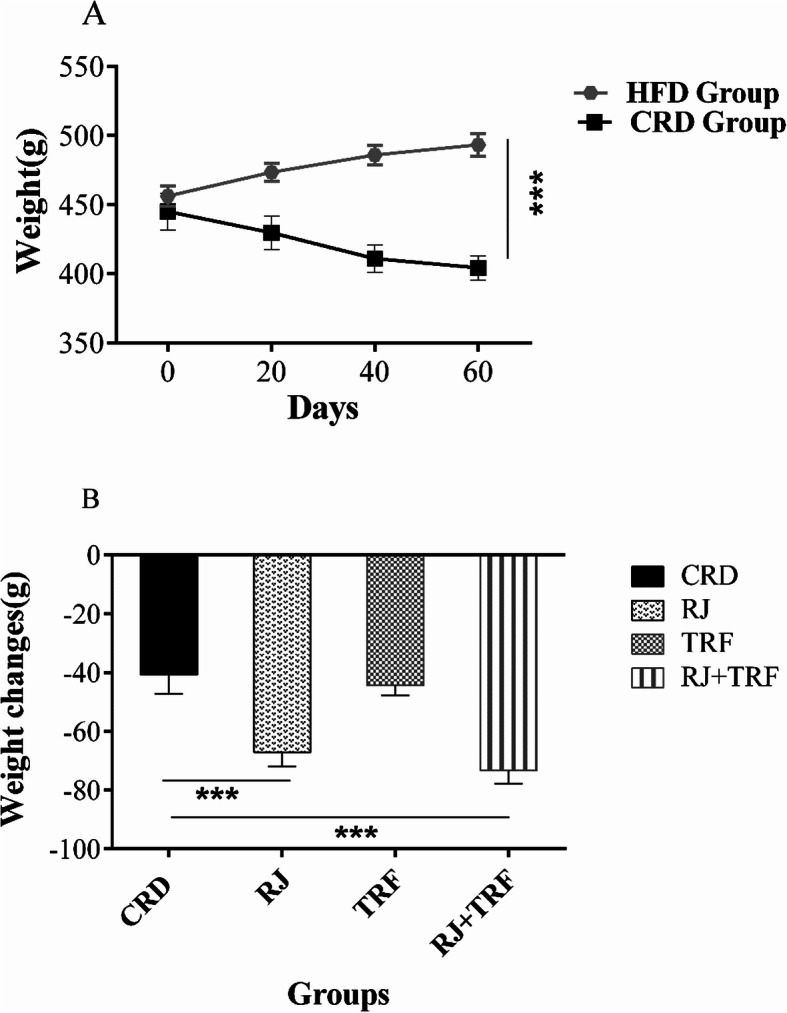
**a** Comparison of body weight after 8 weeks of treatment in the CRD vs. HFD group. **b** Bodyweight changes in RJ, TRF and TRF + RJ groups vs. CRD group. Data are expressed as mean ± SEM, (*n* = 10). ***P < 0.001 by one-way ANOVA

All 55 rats completed the intervention for 8 weeks and included in the analysis. After 8 weeks of the experiment, as expected the final mean body weight of CRD-fed obese rats was significantly lower than that of HFD-fed obese rats. (CRD, 404.24 g ±8.65 g vs HFD, 493.28 g ± 8.23 g, *P* < 0.001) (Fig. 2a). The promoting effects of RJ, TRF and combined interventions in weight changes (relative to baseline weights) of CRD-fed obese rats were depicted in Fig. 2b. RJ treatment decreased the weight of rats (RJ group, − 67.21 g ± 4.84 g vs CRD group, − 40.70 g ± 6.50 g, *p* < 0.001).Moreover, RJ + TRF treatments considerably decreased weight (− 73.29 g ± 4.51 g, *p* < 0.001). However, TRF did not significantly reduce the weight (− 44.40 g ± 3.35 g, *p* ≥ 0.05).

### Effects of RJ, TRF and mixed supplementation on key thermoregulatory genes expressions

**Fig. 3:**
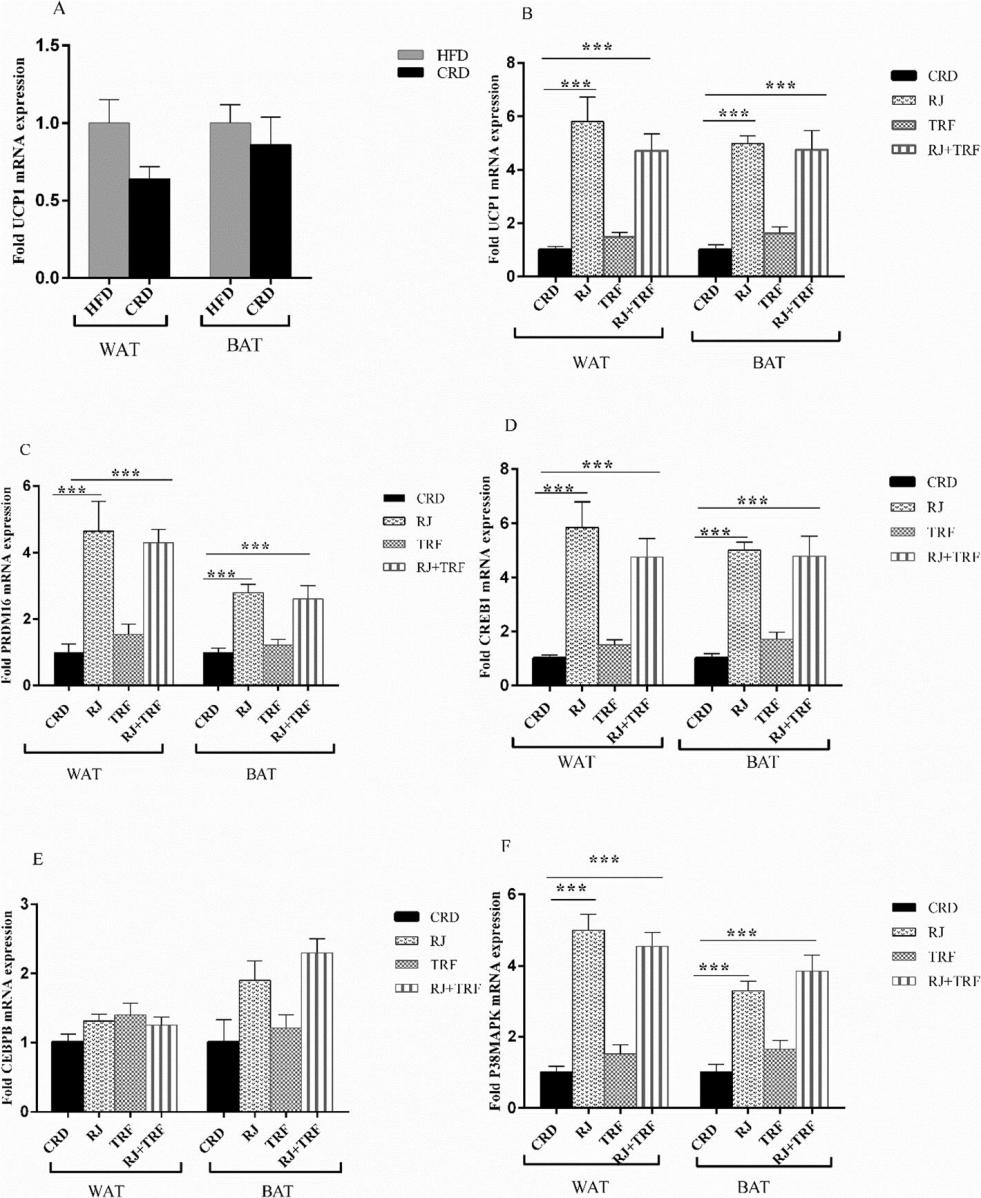
**a**, **b** Effect of CRD after 8 weeks of intervention, on the expression of *UCP1* vs. HFD in WAT and BAT, quantified by qRT-PCR. **c**-**f** Effect of RJ, TRF and TRF + RJ treatment on expression of brown fat-specific and thermogenic genes vs. CRD in WAT and BAT quantified by qRT-PCR. Data are presented as the mean ± SEM (*n* = 10). ***P < 0.001 by one-way ANOVA

To investigate the effects of CRD on thermogenesis, we first measured the expression levels of a key regulatory gene, *UCP1* by RT_PCR in WAT and interscapular BAT. As anticipated, along with weight loss, CRD down-regulated the *UCP1* levels by 36 and 14% in WAT and BAT respectively compare to HDF-fed rats, although there were no statistically significant (*P* ≥ 0.05) (Fig. 3a). We next tested whether RJ and TRF supplementation with CRD would be able to ameliorate the aforementioned effects of CRD on the *UCP1* levels. Our data demonstrated that RJ added in CRD rats led to a significant elevation of *UCP1* in comparison with the CRD-matched group in WAT and BAT as depicted in Fig. 3b (*P* < 0.001). We also observed that TRF induced the *UCP1* levels in both adipose tissues compared to the CRD group, although it was not statistically significant (*P* ≥ 0.05). Furthermore, RJ + TRF significantly induced the *UCP1* levels in comparison to the CRD-matched group (*P* < 0.001). However, the enhancing effects of RJ on *UCP1* expression was superior. Next, we examined the effects of RJ and TRF on key brown fat marker *PRDM16.* As depicted in Fig. 3c mRNA levels of *PRDM16* was increased significantly about 4.65-fold and 2.80- fold in WAT and BAT in the RJ group respectively relative to CRD group (*P* < 0.001). Whereas, TRF did not remarkably up-regulate the *PRDM16* expression in none of the adipose tissues (*P* ≥ 0.05). Moreover, gene expression of *PRDM16* up-regulated significantly in the RJ + TRF group to 4.30 and 2.61 -fold in WAT and BAT respectively in comparison with the CRD group (*P* < 0.001).To further investigate the potential mechanisms underlying the browning effects of RJ and TRF, we determined expression levels of *CREB1* and *CEBPβ,* the master regulators of the thermogenic program. Gene expression of *CREB1* increased significantly by RJ addition to 5.85 and 5 fold relative to CRD group in WAT and BAT respectively (*P* < 0.001).However, TRF treatment did not affect the expression of *CREB1* in comparison with the CRD group (*P* ≥ 0.05). Furthermore, combinations of RJ + TRF markedly increased *CREB1* expression in WAT and BAT relative to the CRD group (*P* < 0.001, Fig. 3d). Furthermore, there were not a significant increase in mRNA levels of *CEBPβ* in any studied groups in WAT and BAT (*P* ≥ 0.05, Fig. 3e).

*UCP1* activation can be regulated by various protein kinases. Therefore, we probed gene expression of *P38MAPK* in above mentioned adipose tissues. Expression of *P38MAPK* increased significantly in the RJ group to 5 and 3.30 -fold in WAT and BAT respectively relative to CRD group (*P* < 0.001).Moreover, TRF did not change the expression of *P38MAPK* notably (*P* ≥ 0.05). Furthermore, the combination of RJ + TRF contributed to a significant increment of the *P38 MAPK* mRNA level in both adipose tissues in comparison with the CRD group (*P* < 0.001, Fig. 3f).

### Effects of RJ, TRF and mixed supplementation on *BMPs* pathway

**Fig. 4:**
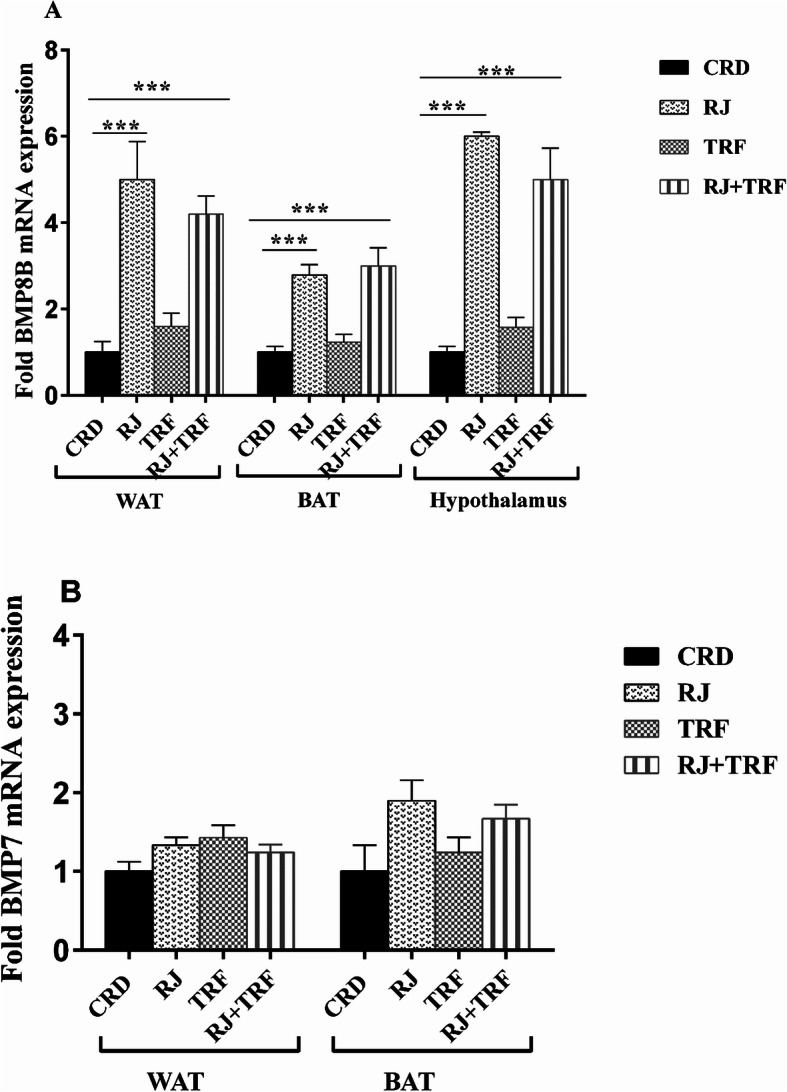
Effect of RJ, TRF and TRF + RJ treatment on the expression of *BMP8B* gene in WAT, BAT**,** and hypothalamus (**a**) and expression of *BMP7* gene in WAT and BAT vs. CRD quantified by qRT-PCR. Data are presented as the mean ± SEM (*n* = 10). ***P < 0.001 by one-way ANOVA

Considering that BMPs are signaling molecules that regulate the thermogenic program and function of classic brown adipose tissue, we measured the expression level of *BMP8B* and *BMP7* in WAT, BAT hypothalamus. Our findings revealed that RJ significantly elevated *BMP8B* expression levels (5, 2.79 and 6 folds) in WAT, BAT, and hypothalamus respectively relative to CRD group (*P* < 0.001, Fig. 4a). Moreover, TRF did not show significant upregulation of *BMP8B* in any of the aforementioned tissues (*P* ≥ 0.05). Intriguingly, RJ + TRF increased *BMP8B* expression level significantly by 4.2, 3 and 5 fold in WAT, BAT and hypothalamus, respectively compared with CRD group (*P* < 0.001).

*BMP7* plays an important role in whole energy homeostasis, adipogenesis, and energy expenditure. However, we founded that RJ, TRF and their combination did not alter the expression of *BMP7* mRNA levels in this study (*P* ≥ 0.05, Fig. 4b).

### Histological results

**Fig. 5:**
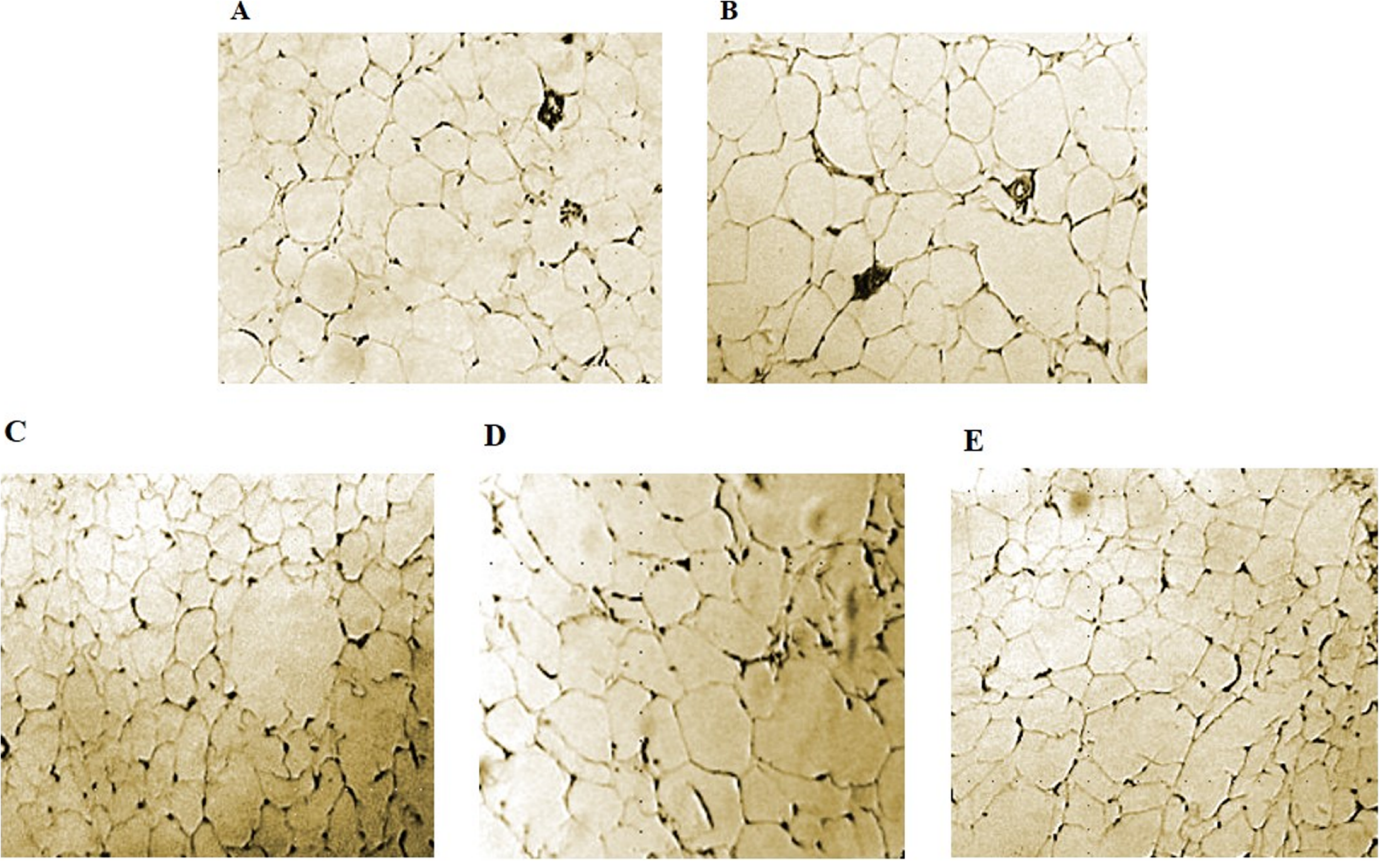
Hematoxylin and eosin (H&E) staining of inguinal WAT section of CRD (**a**), HFD (**b**), RJ (**c**), TRF (**d**) and RJ + TRF (**e**) received rats

As illustrated in Fig. 5a in CRD-fed rats, white adipocytes appeared smaller than HFD-fed rats with unilocular adipocytes (Fig. 5b). There was no evidence of WAT beiging in CRD and HFD –fed rats. Notably in RJ treated group we found small, multilocular beige adipocytes in WAT, (Fig. 5c) Whiles in TRF group WAT changes were not considerable (Fig. 5d). In the RJ + TRF group manifestation of some multilocular adipocytes among white adipocytes was noticed (Fig. 5e).

**Fig. 6:**
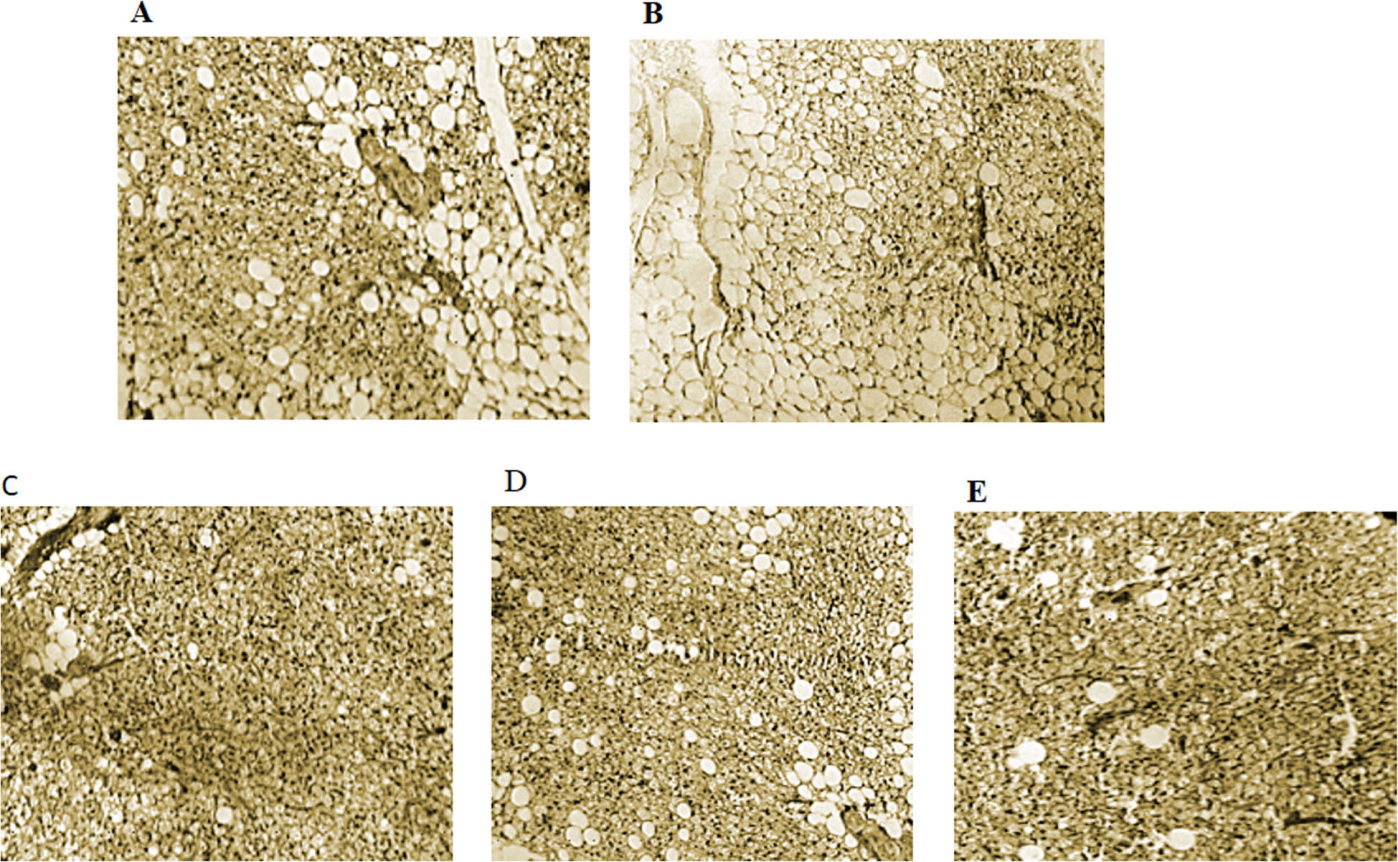
Hematoxylin and eosin (H&E) staining of interscapular BAT section of CRD (**a**), HFD (**b**), RJ (**c**), TRF (**d**) and RJ + TRF (**e**) received rats

Interscapular BAT in the CRD group is observable with some white adipocytes nearby (Fig. 6a), However, in the HFD group, we observed more unilocular white adipocytes along with typical brown adipocytes. (Fig. 6b). In the RJ and RJ + TRF group, BAT is distinguished with more reddish-brown appearance, greater compacted brown adipocytes with multilocular lipid droplets compared to CRD group (Fig. 6c and e), whereas BAT in the TRF group was less compact with white morphology and more connective tissues (Fig. 6d).

## Discussion

Over the last decades, the rapid changes in lifestyle and dietary patterns in modern life bring about the alarming pandemic of obesity. Although Calorie restriction (CR) through confining energy intake is the most common lifestyle dietary intervention to protect against weight gain, it is ineffective in the long-term, since thermogenic adoptions, as defensive mechanisms, diminish the energy expenditure to stop energy depletion [2–4, 27]. Therefore, stimulation of BAT development in WAT (so-called browning) or increasing BAT function to enhance energy expenditure would be considered as promising approaches to manage adiposity. Up to the best of our knowledge, it is the first research investigated the browning and thermogenesis properties of Royal jelly and γ- tocotrienol in the obesity model of rats during CRD. In this study, we demonstrated that, RJ decreased adiposity, induced brite phenotype in WAT, and activated BAT thermogenic program during CRD through a significant up-regulation of *UCP1* as an indicator protein of brown adipocyte concomitant with the increased expression of *PRDM16*; a principal modulator of BAT development; and *P38MAPK, BMP8B, and CEREB1* as other thermogenic components.

During 8 weeks of CRD, we noted insignificant reductions in *UCP1* expression in WAT and BAT concomitant with weight loss about 36 and 14% in comparison to HFD-fed obese rats, respectively. Accordingly, this might be an adaptive response (whitening) to the limited calorie intake or a negative energy balance. However, since the reduction in the *UCP-1* level was not significant, this notion needs more investigations to be approved. Reduction in *UCP1* expression throughout CR is in line with other previously performed studies [2, 3]. However, in the present investigation, due to the short duration of the study, it did not reach a significant level. Therefore, we investigated the molecular changes in thermogenic machinery following the addition of RJ and γ- tocotrienol into CRD-fed rats. Our results showed that, 8 weeks of RJ administration (100 mg/kg/day) to obese rats underwent CRD enhanced the *UCP1* expression levels in both adipocytes. Consistent with these findings, Yoneshiro et al. reported that, 5% lyophilized RJ powder in HFD-induced obese rats enhanced *UCP1* mRNA expression in BAT, but not in WAT without modifying food intake, which suggest the possible augmentation of thermogenesis in BAT and energy expenditure [28]. It was suggested that, RJ attenuated the adverse effects of CR on thermogenesis via increasing BAT activity and WAT remodeling. However, TRF (85 mg/kg/day) added into CRD -fed rats was not efficient in the up-regulation of *UCP1* and also other thermogenic regulators in adipose tissues. However, several studies revealed the positive thermogenic effects of other vitamin E analogs (α-tocopherol and δ tocopherol) in *UCP1* gene induction in WAT of rats and also mouse preadipocytes [29, 30]. In our study, TRF was used comprised of all isoforms of tocotrienols along with α- tocopherol. It is speculated that, α- tocopherol can interact with tocotrienols and represses their activities, and also other isomers of tocotrienol in TRF may produce a synergistic or antagonistic impact with γ- tocotrienol; and therefore, may affect the outcomes. Thus, a pure isomer of γ- tocotrienol may provide different results. In this study, we confirmed that, RJ + TRF combined treatment in CRD obese rats significantly induced the expression of hallmark protein of thermogenesis, *UCP1*, and almost other thermogenic genes mRNA in both adipose tissues. Bearing in mind that, TRF has no remarkable effect on *UCP1* and other regulators of thermogenic program expression, and the *UCP1*s induction is attributed to RJ treatment in the RJ + TRF group. Hence, in the present investigation, we mechanistically explored the thermoregulatory and browning potency of RJ.

Our results show that, RJ added into CRD-fed obese rats caused a significant reduction in body weight to a greater extent related to CRD alone (− 67.21 g ± 4.84 g vs − 40.70 g ± 6.50 g). Accordingly, it is in agreement with Yoneshiro et al.’s study who reported that, 5% of RJ restrained HFD-induced obesity and diminished the white adipose tissue collection in young mice without moderation in food intake [28]. The present study could not demonstrate the remarkable effect of TRF on weight loss of CRD-fed obese rats. Furthermore, Wong et al. reported that, administration of 120 mg/kg/day TRF for 8 weeks did not change body weight gain in HFD-fed Wistar rats [20]. In contrast, in young C57BL/6 J mice, supplementation of HFD with 0.05% γT3 for 4 weeks ameliorated HF diet-mediated obesity [31]. Different genetic backgrounds of the studied animals and supplementation of pure γT3 versus TRF are considered as possible major contributing factors in achieving inconsistent outcomes.

**Fig. 7:**
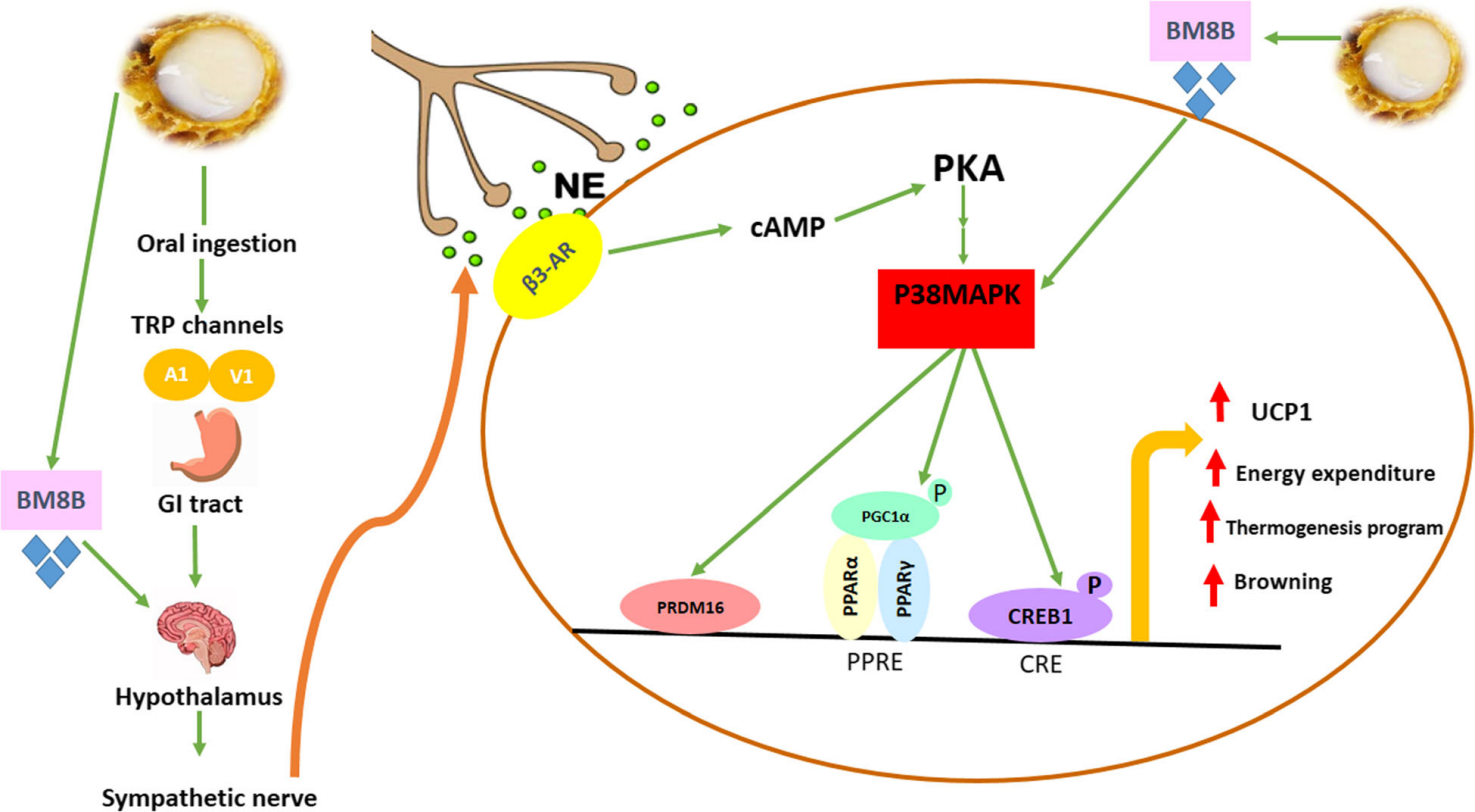
Suggested pathways for browning of white adipose tissue and brown fat activation by RJ. RJ with agonistic activity, activate temperature-sensitive TRP channels in the GI, triggering thermogenesis through the activation of the TRP-SNS-*UCP1* axis. Β3-AR (β3-adrenoceptor), *BMP8B* (bone morphogenetic protein8B), cAMP (cyclic adenosine monophosphate), *CREB1* (cAMP response element-binding protein1), NE (norepinephrine), *PRDM16* (PR domain containing16), *PPARγ* (peroxisome proliferator-activated receptor-gamma), *PGC-1α* (peroxisome proliferator-activated receptor gamma coactivator 1-alpha), PPARα (peroxisome proliferator-activated receptor alpha), *P38 MAPK* (P38 mitogen-activated protein kinase), PKA (cAMP-dependent protein kinase A), TRP (transient receptor potential) and *UCP1* (uncoupling protein 1)

Recent studies found out new transcriptional components such as *PRDM16, C/EBPβ*, and *CEREB1*, which control BAT development and promote brown adipogenesis in inducible WAT. *PRDM16* induces thermogenic program by interacting with peroxisome proliferator-activated receptor-gamma (*PPARγ*) *C/EBP-β*, peroxisome proliferator-activated receptor gamma coactivator 1-alpha (*PGC-1α*), and peroxisome proliferator-activated receptor alpha (*PPARα)* in the regulatory promoter site of the UCP1 gene [8, 32, 33]. Our data revealed that, RJ treatment for CDR-fed obese rats induced *PRDM16* and *CREB1* mRNA levels; however, it did not induce *C/EBPβ* mRNA in both WAT and BAT. It was identified that, HDEA and HDAA, as the main functional compounds in RJ, are responsible for its biological activity [16]. Also, HDEA and HDAA act as agonists of temperature-sensitive Transient receptor potential (TRPs) channels, specially TRPA1 in sensory neurons of the gastrointestinal tract, provoke thermogenesis via a β-adrenergic receptor-mediated pathway in classic brown and inducible white adipocytes, and in addition, simulate cold-induced non-shivering thermogenesis [16].Therefore, in this study, the TRP-SNS-*UCP1* pathway activity is the proposed mechanism for thermoregulatory effects or RJ. Figure 7 demonstrate the suggested molecular mechanisms of RJ effect on thermogenesis induction and browning of WAT. Despite the increasing trends, we found no significant effects of TRF treatment on *PRDM16, C/EBPβ, and CEREB1* induction during 8-week experiment. The reason why the expression of key thermoregulatory components was not remarkably up-regulated by TRF, remains currently unclear; however, the inhibitory effect of α-tocopherol on the absorption of γ-tocotrienol and suppression of its activity, insufficient doses of TRF or limitations of treatment duration may be the reasons. The downstream molecular signaling in the TRP-SNS-*UCP1* pathway mainly includes discharging of norepinephrine from the sympathetic nerve terminals; provoking the tissue that mostly acting through β3-adrenergic receptors; and finally cAMP-dependent protein kinase A (PKA) activation. Phosphorylated PKA leads to the *P38MAPK* phosphorylation, which in turn activates *CREB1* and *PGC1-α* co-activators, and ultimately results in transcription of *UCP1* [32]. Also, the proposed pathways are depicted in Fig. 7. The *P38MAPK* signaling has been defined as one of the important pathways triggering beiging and thermogenic machinery in various models [8]. Our results robustly demonstrated the potency of RJ, but not TRF, to induce the *P38MAPK* mRNA level in white and brown adipose tissues of CRD-fed rats. Therefore, these data support the theory of agonistic activity of RJ at TRPA1 channels in the TRP-SNS-*UCP1* pathway.

*BMPs* are the components of the Transforming growth factorβ (TGF-b) superfamily acting as extracellular signaling proteins and affecting the adipogenesis in WAT and development of BAT. *BMP7* promotes the differentiation of brown preadipocytes to mature brown adipocyte through *PRDM16, PGC-1α* induction, and the increased expression of *UCP1* by *P38MAPK* dependent pathways [13]. *BMP8Bs* are signaling molecules, which are mostly expressed and active in mature BAT and also Central nervous system (CNS).Therefore, *BMP8B* has both central and peripheral actions, which its consonant performance in CNS and BAT can regulate thermogenesis and energy balance through the increased response to noradrenaline in mature BAT and the enhancement of *p38MAPK/CREB* signaling [12, 34]. Moreover, in the brain, *BMP8B* can increase the level of thermogenesis sympathetic activation. Our data demonstrated that, RJ but not TRF added to CRD induced the expression of *BMP8B* in BAT, WAT, and hypothalamus. Although the molecular mechanism by which RJ induced the *BMP8B*, is not known yet, it is suggested that, RJ may cooperate with *BMP8B* to increase the response to noradrenaline in adrenergic receptors and trigger the thermogenic program (Fig. 7). Despite the increment of the *BMP7* mRNA level resulted from RJ treatment, it did not reach a significant level. Also, longer duration of intervention along with more dosages may give rise to considerable results.

## Conclusion

Here, our results suggest that, RJ induced thermogenic gene expression and activation of BAT and brown-like phenotype emergence in WAT, which is called browning or beigeing. Hence, RJ regulates adaptive thermogenesis by increasing the expression of thermogenic genes. Moreover, our data demonstrated that, RJ treatment could lower body weight in comparison with CRD alone, and prevent the thermogenesis decline or even cessation usually occurring in CR.

These findings suggest an important role for RJ in obesity treatment, and moreover, these outcomes expand our vision toward dietary compounds and fat browning factors, and also propose a new approach in the treatment of obesity through the browning process of adipose tissue. It was the first study assessing the effects of RJ and TRF on CRD –fed obese rats; however, there are few limitations on it. We used no genetically modified rats to confirm the involvement of RJ firmly in TRP-SNS-*UCP1* axis. Therefore, further studies using TRPs or UCP-1 Knockout models or performing the treatments with β-adrenergic blockers seem highly desirable to support the proposed pathway. Also, we found no striking effect of TRF on BAT thermogenesis and/or browning of WAT, since all isoforms of tocotrienols and also α-tocopherol are available in TRF. Therefore, interpreting the results would be difficult, due to the possible interactions of tocopherol and tocotrienols. Also, additional studies with single γ-tocotrienol are required to fill these knowledge gaps.

## Data Availability

The datasets used and/or analyzed during the current study are available from the corresponding author on reasonable request.

## Abbreviations

ANOVA: One-way analysis of variance
BAT: Brown adipose tissue
BMPs: Bone morphogenetic proteins
BMP7: Bone morphogenetic protein7
BMP8B: Bone morphogenetic protein8B
CDNA: Complementary DNA
CNS: Central nervous system
CREB1: cAMP response element-binding protein1
CEBPβ: CCAAT/enhancer-binding protein beta
CR: Calorie restriction
CRD: Calorie restriction diet
HFD: High-fat diet
HDEA: 10-hydroxy-Trans- 2-decenoic acid
HDAA: Hydroxydecanoic acid
PKA: Protein kinase A
P38MAPK: P38 mitogen-activated protein kinases
PRDM 16: PR domain containing 16
PBS: Phosphate-buffered saline
PPARγ: Peroxisome proliferator-activated receptor-gamma
PGC-1α: Peroxisome proliferator-activated receptor gamma coactivator 1-alpha
PPARα: Peroxisome proliferator-activated receptor alpha
RJ: Royal Jelly
RT-PCR: Real-time reverse transcription-polymerase chain reaction
TP: Tocopherols
T3: Tocotrienols
TRF: Tocotrienol rich fraction
TRPs: Transient receptor potential
TGF-b: Transforming growth factorβ
UCP1: Uncoupling protein1
WAT: White adipose tissue
